# New Insights on the Effects of Water on Polymer Inclusion Membranes Containing Aliquat 336 Derivatives as Carriers

**DOI:** 10.3390/membranes12020192

**Published:** 2022-02-05

**Authors:** Clàudia Fontàs, Ruben Vera, Enriqueta Anticó, María del Valle Martínez de Yuso, Enrique Rodríguez-Castellón, Juana Benavente

**Affiliations:** 1 Department of Chemistry, University of Girona, 17003 Girona, Spain; ruben.vech@gmail.com (R.V.); enriqueta.antico@udg.edu (E.A.); 2 Laboratorio de Espectroscopía de Rayos-X, Servicios Centrales de Apoyo a la Investigación de la Universidad de Málaga, 29071 Málaga, Spain; mvyuso@uma.es; 3 Departamento de Química Inorgánica, Facultad de Ciencia, Universidad de Málaga, 29071 Málaga, Spain; castellon@uma.es; 4 Departamento de Física Aplicada I, Facultad de Ciencias, Universidad de Málaga, 29071 Málaga, Spain; j_benavente@uma.es

**Keywords:** polymer inclusion membranes (PIMs), X-ray photoelectron spectroscopy (XPS), scanning electron microscopy (SEM), ionic liquids, Aliquat 336, As(V)

## Abstract

Surface characterization of polymer inclusion membranes (PIMs) using the polymers cellulose triacetate and polyvinyl chloride, containing different ionic liquids (ILs) as carriers, has been performed. Three different ILs have been tested: commercial trioctyl methylammonium chloride (Aliquat 336–AlqCl^−^) and two derivatives bearing the counter anion NO_3_^−^ or SCN^−^ (AlqNO_3_ and AlqSCN, respectively). Surface analysis was performed by scanning electron microscopy (SEM) and X-ray photoelectron spectroscopy (XPS) for both dry membranes and PIMs immersed for 4 days in ultrapure water to investigate the effect of the interaction of water with the membrane’s morphology and composition. XPS analysis of the PIMs revealed that immersion in ultrapure water causes a decrease in the atomic concentration percentage (A.C.%) of the specific IL atoms (Cl, S, and N) when compared with dry samples. Moreover, SEM images of the PIMs containing the IL AlqNO_3_ showed an alteration in the morphology of the membrane due to water contact at surface level, whereas no changes were observed at a bulk level. These changes in the surface composition of the water equilibrated PIMs may be associated with the solubilization of the IL in the water solution, which, therefore, may affect the reactivity of the membrane’s surface. To better understand this effect, PIMs containing both AlqCl and AlqNO_3_ as carriers were used for arsenic (V) transport. It was found that AlqCl was the most effective IL and that the effectivity of the PIM on As(V) removal was not affected after five cycles of the membrane’s reuse.

## 1. Introduction

Polymer inclusion membranes (PIMs) have emerged in recent years as a separation technology that is able to provide a highly selective, efficient, and cost-effective process for the removal of pollutants from aqueous matrices [1,2,3]. PIMs, which are usually considered to be a type of liquid membrane [4], basically consist of a polymeric matrix that incorporates a carrier (or extractant) which facilitates the transport of the chemical species of interest from the feed phase to the stripping phase, where the analytes are released. A plasticizer is also sometimes added to improve the mechanical properties of this kind of membrane. However, it has already been proved that the use of ionic liquids (ILs) as carriers favors the plasticity of the membrane, allowing the elimination of plasticizers and, consequently, simplifying the formulation of the PIMs [5].

Ionic liquids (ILs) are low vapor pressure salts of an organic cation and an organic or inorganic anion [6]. They also have limited solubility, good electrical conductivity, and thermal stability, as well as high ion mobility and viscosity [6]. Their low vapor pressure allows ILs to be used in a large number of different applications, such as solvents for organic reactions and catalysis, electrodeposition, extraction, and CO_2_ capture devices [7,8,9]. ILs may be used as substitutes for volatile organic solvents and in new processes, since the variety of possible chemical compositions allows their properties to be tailored to specific applications [10]. ILs have also been considered as good candidates to improve the behavior of membranes for fuel cell applications [11,12,13,14].

Among the different ILs, trioctylmethylammonium chloride (commercially available as Aliquat 336 –AlqCl-^)^ is widely used in the preparation of PIMs, whereas cellulose triacetate (CTA) and polyvinylchloride (PVC) are commonly considered as polymer supports [3,5,15,16,17,18]. However, the great majority of studies using PIMs evaluate their use in transport systems without considering basic aspects regarding the composition of PIMs or solution interactions, despite such information being of significant interest in selecting the most appropriate membrane. In our previous studies [5,19,20], we demonstrated that both physicochemical and electrochemical characteristics of PIMs were affected by the amount of Aliquat 336 content in addition to the nature of the counter-anion (Cl^−^, NO_3_^−^ or SCN^−^). It was established from contact angle measurements that the hydrophobicity of the PIMs with different IL derivatives followed the trend AlqSCN > AlqNO_3_ > AlqCl, which was related to the strength of the ion pair formed between the cationic and anionic part of the IL, and closely related to the stability of the membrane.

Taking into account that the surface of a membrane is the first flow barrier, its characterization is of great importance, since it can significantly influence the properties of the membrane itself. Scanning electron microscopy (SEM), atomic force microscopy (AFM), X-ray photoelectron spectroscopy (XPS), and contact angle measurement are commonly used to determine morphology (dense or porous), structure (symmetric or composite), topography (pore-size distribution), chemical composition, and the wettability of membranes [21,22,23,24]. Other techniques such as tangential streaming potential (TSP), phase-contrast scanning force microscopy (SFM), and spectroscopic ellipsometry (ES) are used for more specific membrane characterization (surface charge, material viscoelastic behavior, or layer thickness in the case of non-uniform or composite membranes) [25,26,27,28]. In particular, XPS is nowadays one of the most popular surface analytical techniques due not only to the exceptional combination of compositional and chemical information, but also to the operational simplicity of this high vacuum technique and the availability of commercial equipment. XPS can be used to determine or confirm the empirical formula of a material and the quantity of the elements present on or near the surface (up to 10 nm of depth) and also for fouling studies in the case of membranes used in different separation processes [26,29].

In this study, both morphological and chemical surface analysis for PIMs prepared with three ILs (commercial AlqCl and the two derivatives, AlqNO_3_ and AlqSCN) are described. XPS investigations of PIMs after water contact were performed and the results are compared with those previously obtained for dry PIMs to estimate possible hydration effects. The effect of the base polymer is also considered by comparing AlqCl–CTA and AlqCl–PVC samples. SEM images were also obtained for pristine PIMs containing the different ILs at different contents in a CTA matrix, and for the PIM containing AlqNO_3_, before and after water contact. Finally, PIMs containing both AlqCl and AlqNO_3_ have been evaluated for the transport of As(V).

## 2. Materials and Methods

### 2.1. Reagents and Solutions

Aliquat 336 was obtained from Sigma-Aldrich (USA), whereas the polymers CTA and PVC were purchased from Fluka (Bern, Switzerland). Chloroform (for CTA) and tetrahydrofuran, THF (for PVC), both from Panreac (Barcelona, Spain), were used to dissolve the polymer and the ILs. NaSCN and NaNO_3_ (Panreac, Barcelona, Spain) were used as the anion source to prepare the Aliquat 336 derivatives.

Stock solution (100 mg L^−1^) of As(V) was prepared from solid Na_2_HAsO_4_·7H_2_O purchased from Merck (Darmstadt, Germany). Working solutions of arsenate in ultrapure water were prepared by dilution of the corresponding stock solution. Sodium chloride, obtained from Fluka (Bern, Switzerland), was used to prepare the stripping solution.

All reagents and solvents were of analytical reagent grade. Ultrapure water obtained from a Milli-Q Plus water purification system (Millipore Ibérica, Madrid, Spain) was used.

### 2.2. IL Preparation

**Figure 1:**
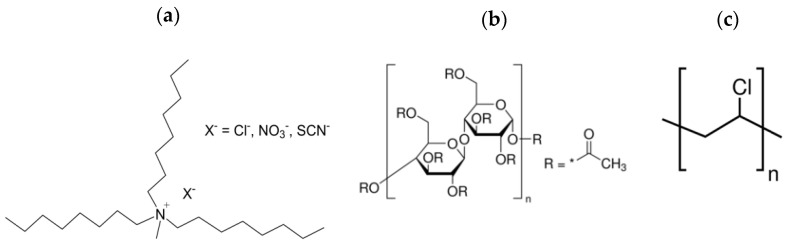
Chemical structure of: (**a**) ILs; (**b**) cellulose triacetate (CTA); and (**c**) polyvinyl chloride (PVC).

Commercial Aliquat 336 was used for the preparation of the two other ILs, AlqNO_3_ and AlqSCN, by exchanging the chloride anion present in the formulation of Aliquat 336 by nitrate or thiocyanate anions, respectively (see their chemical structure in Figure 1a) [19]. For this, 5 g of Aliquat 336 were transferred into a separation funnel and dissolved in 50 mL of chloroform. Different sodium salts (NaNO_3_ and NaSCN) were slowly added to the solution in an amount that was 30% greater than the amount of Aliquat 336. The mixture was vigorously stirred for 5 h at room temperature. Afterwards, it was rinsed with water to remove both the excess sodium salt as well as the sodium chloride that was formed. Washing was repeated until no chloride was detected in the water sample (by the addition of silver nitrate) and the organic solution was dried with magnesium sulphate. Finally, chloroform was evaporated under reduced pressure until a brownish viscous liquid was obtained.

### 2.3. PIMs Preparation

Preparation of PIMs using cellulose triacetate (CTA) or polyvinyl chloride (PVC) as base polymers (see Figure 1b,c for the chemical structure of the polymers) was previously detailed [5,19]. Briefly, 200 mg of CTA or 400 mg of PVC were dissolved in 20 mL of chloroform or in THF, respectively; then, the corresponding amount of the ionic liquid was added and magnetically stirred for 2 h. Finally, the solution was poured into a 9.0 cm diameter flat bottom glass Petri dish, which was set horizontally and loosely covered. The solvent was allowed to evaporate overnight at room temperature and the resulting film was peeled off the Petri dish.

In this study, membranes with different ILs:polymer ratios have been used to reveal specific changes or effects more clearly. The amount of IL incorporated in the PIM is described in % (mass), and the content was 60% and 30% for the characterization studies for all three ILs in CTA. Membranes with AlqCl and PVC were also investigated. Consequently, the resulting membranes were named as follows: (60%AlqCl, 60%AlqNO_3_, or 60%AlqSCN)–40%CTA and 60%AlqCl–40%PVC and (30%AlqCl, 30%AlqNO_3_, or 30%AlqSCN)–70%CTA. Furthermore, a PIM made of 50%AlqNO_3_–50%CTA was also prepared for the investigation of the effect of water using SEM and for transport studies.

### 2.4. XPS Measurements

In order to evaluate the effect of water contact on the surface of the membrane, pieces of the PIMs were submerged in ultrapure water for 4 days (50 mL of ultrapure water and a piece of membrane of 4 cm^2^). These PIMs were labelled as “water equilibrated” samples and identified with the letter w, whereas dry samples (pristine PIMs) were identified by the letter d.

Chemical surface characterization of the PIMs was performed by a Physical Electronics spectrometer (PHI 5700) with X-ray Mg K_α_ radiation (300 W, 15 kV, 1253.6 eV) as the excitation source. High-resolution spectra were recorded at a given take-off angle of 45° by a concentric hemispherical analyzer operating in the constant pass energy mode at 29.35 eV, using a 720 μm diameter analysis area. Accurate ±0.1 eV binding energies were determined with respect to the position of the adventitious C *1s* peak at 285.0 eV, and the residual pressure in the analysis chamber was maintained below 5 × 10^−7^ Pa during data acquisition.

Membranes were mounted on a sample holder and kept overnight at high vacuum in the preparation chamber before being transferred to the analysis chamber of the spectrometer for testing. When analyzing the pure IL, a drop was deposited on a glass slide. Each spectral region was scanned several times until a good signal-to-noise ratio was observed. The PHI ACCESS ESCA-V6.0 F software package was used for acquisition and data analysis. A Shirley-type background was subtracted from the signals. Recorded spectra were always fitted using Gauss–Lorentz curves to accurately determine the binding energy (BE) of the different element core levels, as has previously been described in detail [30]. Atomic concentration percentages (A.C.%) of the characteristic elements found on the surfaces of the analyzed samples were determined, taking into account the corresponding area sensitivity factor [30] of the different measured spectral regions.

### 2.5. Scanning Electron Microscopy

SEM images were performed with a scanning electron microscope FE-SEM Hitachi, S-4100 (Tokyo, Japan), and the samples were placed on a stub and coated with carbon (K950 turbo evaporator, Emitech, Germany). For cross-section observations, the membranes were frozen and broken under liquid nitrogen. Digital images were collected and processed by the Quartz PCI software. This technique was used to obtain micrographs of the different PIM formulations as well as of dry and water-equilibrated membranes (after being immersed in ultrapure water) in the case of the PIM made of 50%AlqNO_3_–50%CTA.

### 2.6. Transport Experiments for As(V)

Transport experiments were carried out using both 50%AlqNO_3_–50 CTA and 50%AlqCl–50%CTA membranes in a two-compartment permeation cell containing 190 mL of both feed (consisting of 10 mg L^−1^ As at pH 7) and stripping (0.1 M NaCl) solutions, well stirred, and a PIM with an exposed area of 11.5 cm^2^. Samples from feed and stripping solutions were taken after 24 h for subsequent metal content analysis. All experiments were carried out at room temperature of 22 ± 1 °C. Transport efficiency (TE) of As was determined by using Equation (1):

(1) $$TE\left(\%\right)=\frac{\left[\mathrm{As}\left(V\right)\right]\mathrm{strip}\left(t\right)}{\left[\mathrm{As}\left(V\right)\right]\mathrm{feed}\left(0\right)}\times 100$$

where [As(V)]_strip(t)_ denotes As concentration in the stripping compartment at an elapsed time t and [As(V)]_feed(0)_ is the initial As concentration in the feed phase. An inductively coupled plasma emission spectrometer (ICP-AES) (Liberty RL, Varian Australia, Mulgrave, Vic., Australia) was used for the analysis of arsenic in the feed and stripping phases at 228.8 nm.

## 3. Results and Discussion

### 3.1. Chemical Surface Characterization of PIMs by XPS

Taking into account that PIMs are commonly used in separation processes for analytes present in aqueous solutions, it is of interest to establish possible chemical changes associated with water–IL–polymer interactions. In fact, the XPS technique has been employed for the characterization of PIMs prepared with the IL Aliquat 336 and its derivatives in dry state in earlier studies [5,19]. Table 1 shows the average A.C.% values (determined from measurements performed on both faces of the membranes) of the elements found on the water-equilibrated PIMs surfaces. For comparison purposes, data for dry state from [19] are also included. Common atoms in all PIMs made of CTA are, on the one hand, N (from the cationic part of the IL –trioctyl metil ammonium cation-) and, on the other hand, O and C, both from the polymer and/or the IL. A certain percentage of silicon, a non-characteristic atom which did not originate from either the IL or the polymer, was also detected, which is associated with SiO_2_ and attributed to the membrane preparation. Focusing on the A.C.% of the common atoms, it can be observed in Table 1 that water-equilibrated samples show lower C% than dry ones, resulting from the solubilization of the components of the membrane containing aliphatic carbon [31,32]. In the case of O% and Si%, the effect is the opposite, since their content is higher for PIM-w samples. This fact can be associated with a more superficial re-orientation of the hydrophobic silicon compounds in water-equilibrated samples, as was found in the analysis of regenerated cellulose membranes reported in [33].

In order to better evaluate the changes caused by water contact to the surface of the different PIMs, we need to take into account the changes in atomic content (%) of the specific anions: chlorine in the case of the PIM 60%AlqCl–40%CTA and sulphur for the PIM 60%AlqSCN–40%CTA. It is important to bear in mind that PIMs containing AlqNO_3_ do not possess a specific atom characteristic of the IL, since N is present in both the anion and the cation of the IL. Moreover, chlorine is not a suitable atom to evaluate the possible changes in the PIM made of PVC, since Cl is also present in both the polymer and the IL.

As can be observed in Table 1, chlorine is not present in any PIMs containing either AlqSCN or AlqNO_3_, confirming that a complete anion exchange has taken place in the preparation of these derivatives. Focusing on the PIM 60%AlqCl–40%CTA, a reduction of 40% of chlorine was found when compared with the dry sample. The reduction of S for PIM 60%AlqSCN–40%CTA was 36%, slightly lower than the value for the PIM with AlqCl. These results show a significant loss of IL on the surface of the PIMs.

In order to estimate changes to the membrane surface associated with the hydration state for 60%AlqNO_3_–40%CTA and 60%AlqCl–40%PVC membranes, it is necessary to analyze core lever signals of N and Cl, respectively.

**Figure 2:**
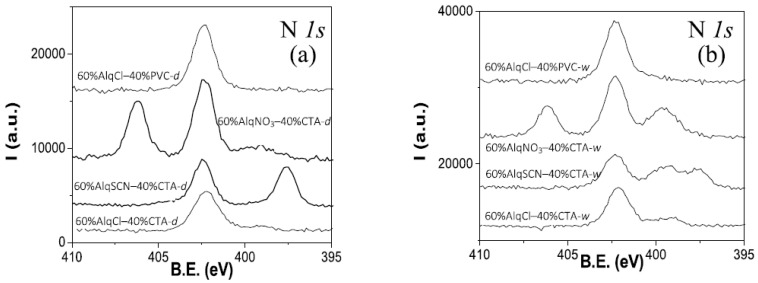
N *1s* core level spectra for dry (**a**) and water-equilibrated (**b**) samples of the studied PIMs.

**Figure 3:**
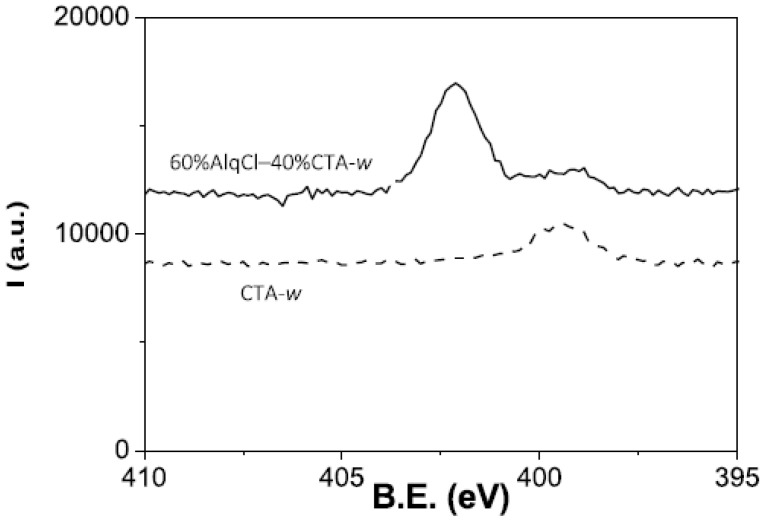
N *1s* core level spectra for water-equilibrated samples of 100% CTA and a 60% AlqCl–40% CTA PIM.

Figure 2 shows the comparison of N *1s* core level signals obtained for dry (Figure 2a) and water-equilibrated (Figure 2b) samples of the four membranes that were studied. As can be observed, the spectra show different signals depending on which IL is present in the membrane, but also a new peak at a B.E. at approximately 399.5 eV. To evaluate the origin of this new peak, a film made only of the polymer CTA was also immersed in ultrapure water and the XPS spectrum was recorded. This spectrum is presented in Figure 3 (100% CTA-w) together with the spectrum of the 60% AlqCl–40% CTA-w PIM, for comparison purposes. As can be seen, the new peak is due to the polymer, and might be associated with the hydration of impurities in the CTA matrix.

The percent areas and corresponding A.C.% of the different nitrogen contributions were obtained by deconvolution of the spectra shown in Figure 2, and their values are indicated in Table 2. N_1_ at 406.4 eV corresponds to NO_3_^−^ anion; N_2_ at 402.4 eV to the N of the cationic part of the IL; and N_3_ at 397.7 eV to SCN^−^ anion. In all cases, a clear reduction in the areas of nitrogen contributions exists when dry and water-equilibrated samples of membranes are compared. Taking into account the values indicated in Table 1 and Table 2, the following ratios for the water-equilibrated samples were obtained: N_2_/Cl = 1.1 (60%AlqCl–40%CTA-w membrane), N_2_/S = 0.94 and N_2_/N_3_ = 1.9 (60%AlqSCN–40%CTA-w), and N_2_/N_1_ = 1.9 (60%AlqNO_3_–40%CTA-w); however, these last two ratios would be reduced if the new peak contributions (at 399.5 eV) were also added (N_2_/N_3_^+^new peak = 0.9 and N_2_/N_1+_new peak = 1.1), in agreement with the compounds’ stoichiometry. Moreover, a reduction of approximately 21% in the percentages of N_1_ for water-equilibrated 60%AlqNO_3_–40%CTA when compared with dry samples was obtained, which demonstrates the greater stability of this PIM formulation (in the case of PIMs with AlqCl and AlqSCN the reduction was 40% and 36%, respectively, as stated above).

**Figure 4:**
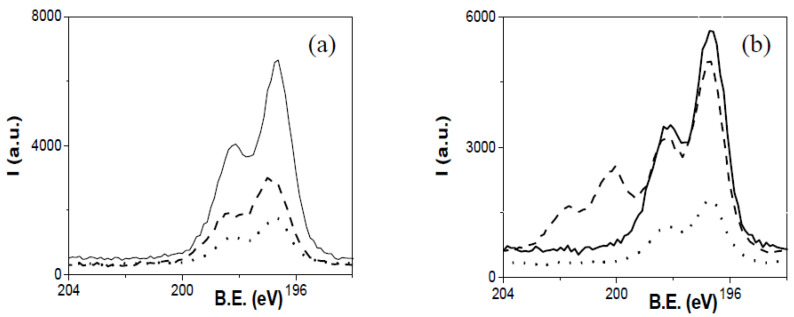
Cl *2p* core level spectra for (**a**) 60%AlqCl–40%CTA-d (solid line) and 60%AlqCl–40%CTA-w (dashed line); (**b**) 60%AlqCl–40%PVC-d (solid line); and 60%AlqCl–40%PVC-w (dashed line). Dotted line in (**a**,**b**) corresponds to pure IL AlqCl.

Figure 4 shows the Cl 2*p* spectrum for dry and water-equilibrated samples of the two PIMs obtained with AlqCl and the two polymers (60%AlqCl–40%CTA (Figure 4a) and 60%AlqCl–40%PVC (Figure 4b)). Both dry samples as well as the 60%AlqCl–40%CTA-w membrane seem to be completely covered by the ionic liquid (which is also represented in Figure 4 by a dotted line), and they show the typical signal for ionic chlorides [34]; however, upon hydration of the PVC-based sample, the presence of the covalent chloride of PVC is clearly observed in Figure 4b. These results provide extra evidence that the hydration process removes part of the AlqCl IL from the PIM surfaces, leaving the polymer structure exposed.

**Figure 5:**
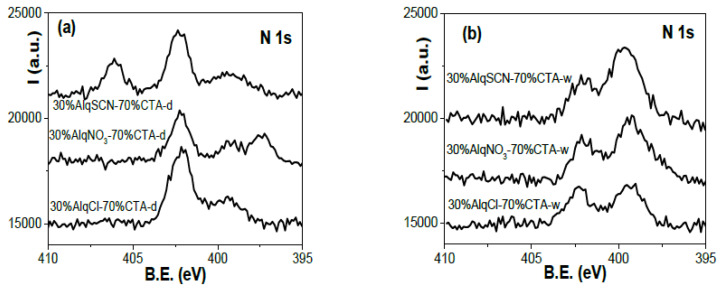
N *1s* core level spectra for 30%AlqCl–70%CTA, 30%AlqNO_3_–70%CTA, and 30%AlqSCN–70%CTA PIMs. (**a**) Dry samples and (**b**) water-equilibrated samples.

Similar results were obtained for PIMs with 30% of IL. Figure 5 shows a comparison of nitrogen core level spectra for 30%AlqCl–70%CTA, 30%AlqNO_3_–70%CTA, and 30%AlqSCN–70%CTA PIMs. Due to the higher CTA content of these membranes, the peak at ~399.5 eV is clearly observed even in dry samples, with the water-equilibrated samples contributing more nitrogen.

Consequently, the XPS analysis performed with water-equilibrated samples of different PIMs demonstrates a certain loss of the ILs used for membrane fabrication, in agreement with reported results obtained from weight loss measurements [35,36], but also the modifications of the PIMs associated with water contact, which depend on both the IL and the polymer.

### 3.2. Morphological Characterization with SEM

**Figure 6:**
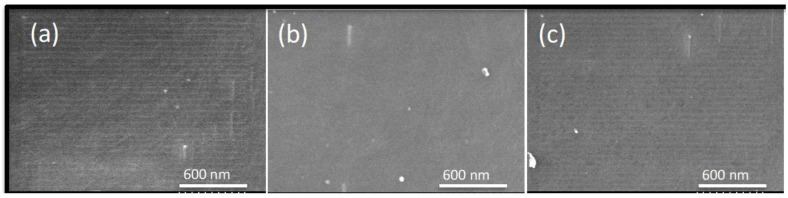
Surface SEM images of dry PIMs with a composition of 30%IL–70%CTA. (**a**) AlqCl; (**b**) AlqNO_3_; and (**c**) AlqSCN.

Possible PIM modifications depending on the type of IL, the concentration, and the contact with water were investigated using SEM. It is significant to note the importance of IL content on the mechanical stability and manageability of PIMs, since brittle samples are obtained when low IL content is used (less than 20%, approximately), but the plasticity of membranes at high IL percentages (higher than 70%) may be too great [5]. It was anticipated that the IL would be physically inserted within the polymer chains, giving a nano-scale structuration, as reported by other authors [37]. As expected, the dry membranes obtained were dense and with no apparent porosity, as can be observed in the SEM micrographs for the PIMs with the lowest IL concentration (i.e., 30%) (Figure 6).

**Figure 7:**
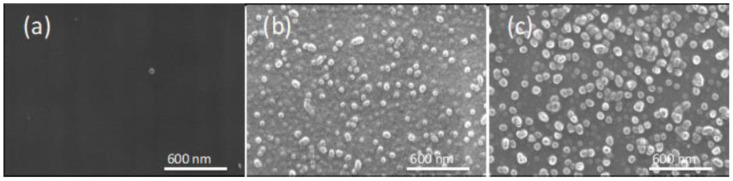
Surface SEM images of dry PIMs with a composition of 60%IL–40%CTA. (**a**) AlqCl; (**b**) AlqNO_3_; and (**c**) AlqSCN.

However, in membranes with a higher IL content (60%), the morphology of the PIMs varies depending on the IL incorporated (see Figure 7). As can be observed in Figure 7a, in the case of AlqCl, the characteristics of the PIM are similar to those of the PIM containing 30% of IL. However, in the case of both PIMs that include the derivatives AlqNO_3_ and AlqSCN, they present a surface with nano-spheres that are uniformly distributed along the surface. The presence of such small nano-spheres can be attributed to the formation of ionic aggregates of IL due to poorer miscibility and/or dispersion of these derivatives in the matrix. The formation of such ionic aggregates in the presence of an excess of IL has previously been determined by other authors [38] and confirmed by dynamic mechanical analysis [39].

**Figure 8:**
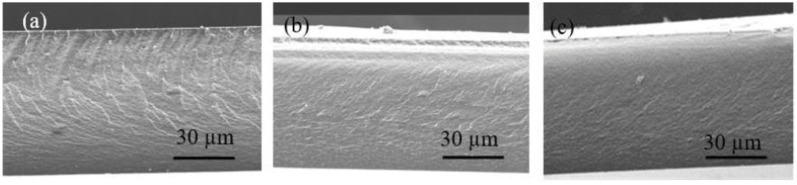
Cross-section SEM images of dry PIMs with a composition of 60%IL–40%CTA. (**a**) AlqCl; (**b**) AlqNO_3_; and (**c**) AlqSCN.

Interestingly, the cross-section images (see Figure 8) show that the morphology of the bulk membrane is completely homogeneous, without the presence of aggregates of the IL, thus demonstrating the significance of the surface characterization.

Importantly, from our results, we can hypothesize that the miscibility of the IL with the polymer is lower for AlqNO_3_ and AlqSCN than for AlqCl. Other authors have pointed to the existence of a miscibility limit between the pre-polymer and the ionic liquid in an epoxy matrix [37,39].

**Figure 9:**
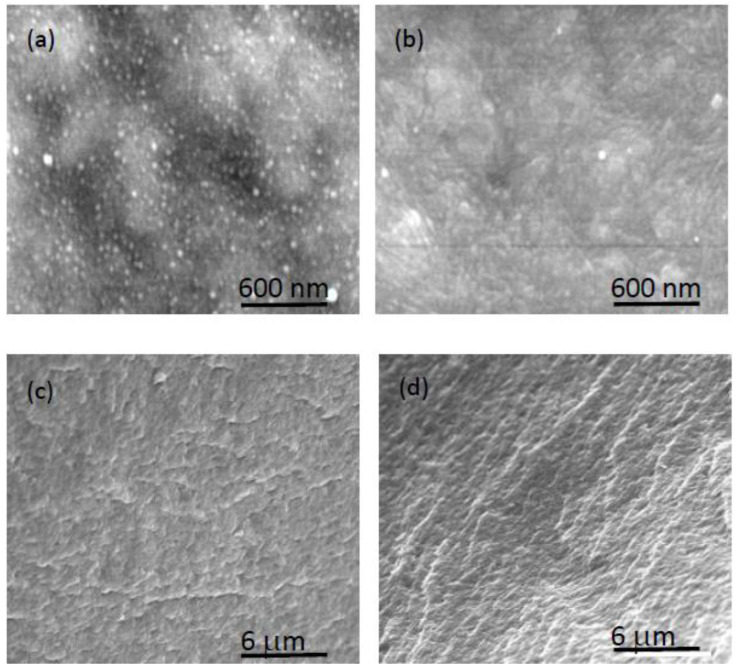
SEM images from a pristine 50%AlqNO_3_–50%CTA PIM: surface (**a**) and cross-section (**c**). The same membrane after immersion in ultrapure water: surface (**b**) and cross-section (**d**).

As it was previously indicated, the formulations used for PIM characterization by XPS and SEM techniques (60% IL/40% polymer or 30%IL/70% polymer) were selected to highlight IL or polymer characteristics, as the PIMs were almost at the limit of adequate mechanical properties. Consequently, a PIM containing an intermediate amount of AlqNO_3_ (50%AlqNO_3_–50%CTA) was also studied, and the results are shown in Figure 9. SEM images were recorded before and after immersion of the PIM in ultrapure water, and both the surface and cross-section images were analyzed. As can be observed, the nano-spheres in the surface of the dry PIM disappear when the PIM is contacted with water. A comparison of cross-section images for water-equilibrated and dry samples shows practically no modification of the membrane bulk. Consequently, it seems that water contact causes morphological changes to the surface of PIMs but not to the bulk part.

### 3.3. Comparision of IL Performance in Transport Experiments

Taking into account the results from the characterization of the membranes, we wanted to investigate whether the stability of the membrane was related to its effectivity as a separation system. For that, we chose the transport of As(V) as the case study. The extraction and transport of arsenic species by means of AlqCl incorporated in a membrane have been successfully reported in the literature [3,40]. However, arsenic transport has not been investigated using the nitrate derivative as a carrier. At neutral pH (∼7), the arsenate ion is present mainly as H_2_AsO_4_^−^ and HAsO_4_^2−^, which are in equal proportion in the solution. This fact allows the As(V) species to be extracted through an ion-exchange mechanism according to the following stoichiometric equation:

HAsO_4_^2−^ + 2(AlqX^−^) ⇋ [(Alq^+^)_2_HAsO_4_^2−^] + 2X^−^ (2)

where Alq^+^ represents the cationic part of Aliquat 336 molecule and X^−^ represents the counter-anion chloride or nitrate.

An alternative which reverses this extraction reaction can be based on the use of chloride at high concentration, as it is expected that arsenic species will be released due to the dissociation of the ion pair formed in the organic phase.

Therefore, we evaluated PIMs (average thickness of 60 μm) consisting of 50% CTA as the base polymer and 50% AlqCl or AlqNO_3_ as the carriers; the first one is the IL that is easily released from the PIM surface, while the second is the IL that provides more stable membranes.

**Figure 10:**
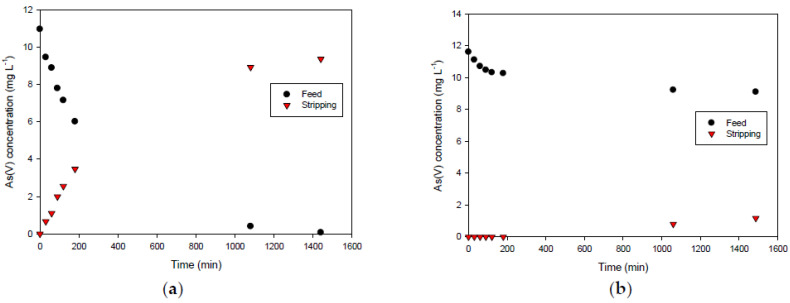
Transient concentration curves in As(V) transport experiments involving 50%CTA–50%AlqCl (**a**) and 50%CTA–50%AlqNO_3_ (**b**). (Experimental conditions: feed solution: 10 mg L^−1^ As(V), pH = 7; stripping solution: 0.1 M NaCl in (**a**) and 0.1 M NaNO_3_ in (**b**)).

The transient As(V) concentration profiles in both the feed and the stripping solutions are shown in Figure 10. As can be seen, the more stable PIM (made of CTA and AlqNO_3_) results in the least efficiency in terms of As(V) transport. This fact may be due to the great interaction between Alq+ and the anion NO_3_^−^, which form a stable ionic pair. Therefore, nitrate is hardly exchanged by arsenate, resulting in poor transportation (about 10%). However, in the case of the PIM containing AlqCl, despite the possible alteration on the surface due to the IL loss, it results in a very effective membrane to remove arsenate.

**Figure 11:**
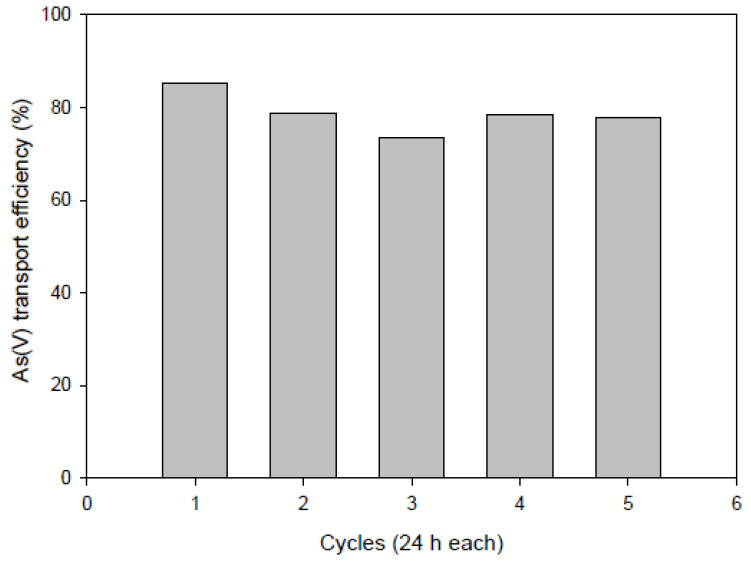
Transport efficiency of the PIM system after five cycles of 24 h each. Feed solution: [As(V)] = 10 mg L^−1^, pH 7. Stripping solution: 0.1 M NaCl.

Moreover, to examine the possible long-term stability of PIMs, we used the same membrane (50%CTA–50%AlqCl) repeatedly in successive experiments transporting As(V) from the aqueous solution at the same concentration (5 cycles of 24 h each). The results obtained in terms of As(V) transport efficiency are plotted in Figure 11 and it can be seen that only slightly variations were measured, with average transport efficiency of <TE> = (79 ± 4)%, without signs of structural weakening.

Then, we can presume that, for the particular formulation of the PIM tested, surface modification does not have a dramatic effect on the transport efficiency since the bulk concentration of the IL is sufficient to ensure the transportation. However, in other systems where the surface of the membrane has an active role, for example when the chemical reaction taking place in the interface acts as a limiting factor or when the membrane itself acts as a catalyst, the modifications occurring at the membrane surface, such as those determined by XPS analysis, could affect the transport, which helps us to understand the behavior of the system.

## 4. Conclusions

Surface characterization by XPS and SEM techniques for different polymer inclusion membranes has been performed with the samples in dry and water-equilibrated states to estimate the possible loss of IL and/or their chemical surface modifications depending on both IL-anion and polymer support. The PIMs studied here were fabricated with three ILs, AlqCl, AlqNO_3_, and AlqSCN, as carriers and cellulose triacetate (CTA) or polyvinyl chloride (PVC) as the support matrix at two different formulations (60% IL-40% polymer or 30%IL-70% polymer). XPS results show the partial loss of the IL in the water-equilibrated samples, which is more significant for the AlqCl–CTA PIM; approximately 40% is calculated from the deconvolution of the XPS spectrum. PIMs made of AlqNO_3_-CTA show the lower loss of the IL from the surface. The nature of the IL-anion also affects the structuration of the polymer as observed in SEM images. It was observed that AlqCl is more miscible with the polymer matrix, whereas, for the other anions, nano-spheres are formed on the surface of the PIM while no changes at bulk level are observed. These nano-spheres disappear upon the PIM coming into contact with water. Moreover, the 50%AlqCl-50%CTA membrane shows good transport efficiency to As(V) (approximately 80%) as well as adequate stability. This latter point was established by considering PIM reusability studies, which demonstrate that the PIM does not practically modify its transport ability after 5 days in contact with aqueous solutions.

## Figures and Tables

**Table 1:** Average values of the A.C.% of the elements found on the surfaces of water-equilibrated (w) and dry (d) PIMs (data for dry state from [20]). *nd: not detected*.

PIM Composition		<C> (%)	<O> (%)	<N> (%)	<Cl> (%)	<S> (%)	<Si> (%)
60%AlqCl–40%CTA	w	78.6 ± 1.9	16.5 ± 0.4	1.8 ± 0.2	1.5 ± 0.1	*nd*	1.3 ± 0.8
	d	86.2 ± 0.8	8.2 ± 1.1	2.7 ± 0.1	2.5 ± 0.2	*nd*	0.4 ± 0.2
60%AlqNO_3_–40%CTA	w	76.3 ± 2.8	15.4 ± 0.7	4.1 ± 0.3	*nd*	*nd*	2.6 ± 1.2
	d	81.1 ± 1.3	13.5 ± 0.9	4.0 ± 0.2	*nd*	*nd*	1.2 ± 0.6
60%AlqSCN–40%CTA	w	78.3 ± 2.1	14.1 ± 0.8	3.4 ± 0.4	*nd*	1.6 ± 0.3	1.7 ± 0.9
	d	86.0 ± 1.5	6.2 ± 1.1	4.5 ± 0.3	*nd*	4.5 ± 0.3	0.8 ± 0.4
60%AlqCl–40%PVC	w	89.7 ± 0.5	2.4 ± 0.5	2.8 ± 0.1	4.0 ± 0.1	*nd*	0.7 ± 0.3
	d	89.0 ± 0.3	4.0 ± 0.3	3.1 ± 0.1	3.7 ± 0.1	*nd*	0.2 ± 0.1

**Table 2:** Area percentages (between brackets in italics) and A.C.% after deconvolution of nitrogen signals obtained for dry and water-equilibrated PIMs.

PIM Composition		N_1_, 406.4 eV		N_2_, 402.4 eV		N_3_, 397.7 eV	
60%AlqCl–40%CTA	d			(91%)	2.5%		
	w			(86.0%)	1.6%		
60%AlqNO_3_–40%CTA	d	(35.1%)	1.4%	(55.4%)	2.2%		
	w	(27.2%)	1.1%	(50.5%)	2.1%		
60%AlqSCN–40%CTA	d			(50.7%)	2.3%	(44.1%)	2.0%
	w			(45.4%)	1.6%	(25.1%)	0.85%
60%AlqCl–40%PVC	d			(100%)	3.1%		
	w			(100%)	2.8%		
